# Successfully treated advanced esophageal cancer with left axillary lymph node metastasis and synchronous right breast cancer: a case report

**DOI:** 10.1186/s40792-015-0102-9

**Published:** 2015-10-06

**Authors:** Yuji Akiyama, Takeshi Iwaya, Yoshihiro Shioi, Fumitaka Endo, Kazushige Ishida, Masahiro Kashiwaba, Koki Otsuka, Hiroyuki Nitta, Keisuke Koeda, Masaru Mizuno, Yusuke Kimura, Akira Sasaki

**Affiliations:** Department of Surgery, Iwate Medical University School of Medicine, Iwate, Japan; Department of Palliative Care Medicine, Iwate Medical University School of Medicine, Iwate, Japan

**Keywords:** Esophageal cancer, Synchronous double cancer, Axillary lymph node metastasis, DCF therapy

## Abstract

The incidence of double cancer of the esophagus and breast is rare, and axillary lymph node metastasis (ALM) in esophageal cancer is also very rare. We report a case of advanced esophageal cancer with left ALM and synchronous right breast cancer. A 64-year-old woman was admitted to our hospital with dysphagia. The clinical diagnosis was esophageal cancer (T3N0M1 stage IV) and right breast cancer (T1cN0M0 stage I). She was initially treated with triple chemotherapy with docetaxel, cisplatin, and 5-fluorouracil. The primary lesion in the esophagus achieved almost complete response as assessed by esophageal endoscopy. A computed tomography scan showed that the left ALM reduced in size and that stable disease was achieved for the right breast cancer. She underwent partial mastectomy of the right breast and bilateral axillary lymph node dissection. The histopathological diagnosis of the breast cancer was T1cN1M0 stage IIA. The lymph nodes from the left axilla contained metastatic cells from the squamous cell carcinoma of the esophagus. Complete response was achieved for the primary lesion in the esophagus following chemoradiotherapy (CRT), and the patient has been relapse free 2 years after treatment. Thus, we report the successful treatment of synchronous double cancers of the esophagus with left ALM and right breast by combination therapy with chemotherapy, CRT, and surgery.

## Background

The incidence of multiple cancers of the esophagus and other organs reportedly ranges from 9.5 to 20.7 % [1]. Head and neck squamous cell carcinoma and gastric adenocarcinoma are most frequently observed as multiple primary cancers of other organs in patients with esophageal cancer [2, 3]. In cases of synchronous multiple cancers with distant metastasis (liver, pulmonary, and/or distant lymph node metastasis), it is sometimes difficult to diagnose the primary site of the distant metastasis and to decide on the order of priority of treatment among the synchronous cancers. The incidence of double cancer of the esophagus and breast is rare [4], and axillary lymph node metastasis (ALM) from esophageal cancer [5] and contralateral ALM from breast cancer [6, 7] are also very rare. Here we report a case of synchronous double cancers of the esophagus with left ALM and right breast.

## Case presentation

**Fig. 1:**
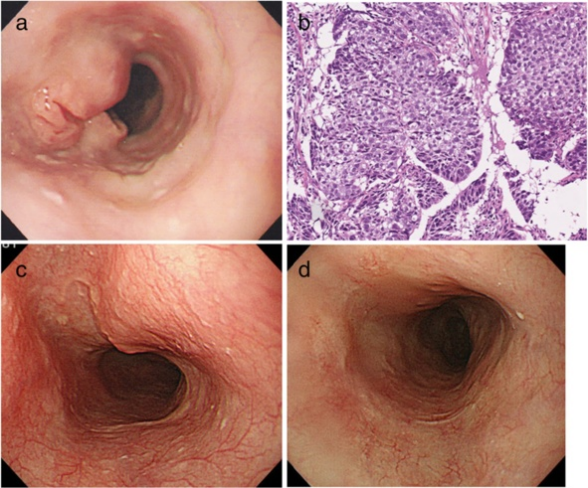
Esophagogastroscopy findings of the esophagus and pathological findings for the esophageal cancer. **a** Esophagogastroscopy revealed an ulcerated circumferential mass in the middle thoracic esophagus. **b** Pathological examination of the biopsy from the esophagus showed squamous cell carcinoma (×100 magnification). **c** Esophagogastroscopy after four courses of chemotherapy with docetaxel, cisplatin, and 5-fluorouracil revealed that the lesion was markedly flattened and only the ulcer was left. **d** Esophagogastroscopy after chemoradiotherapy revealing that the lesion had vanished and only a scar was left

**Fig. 2:**
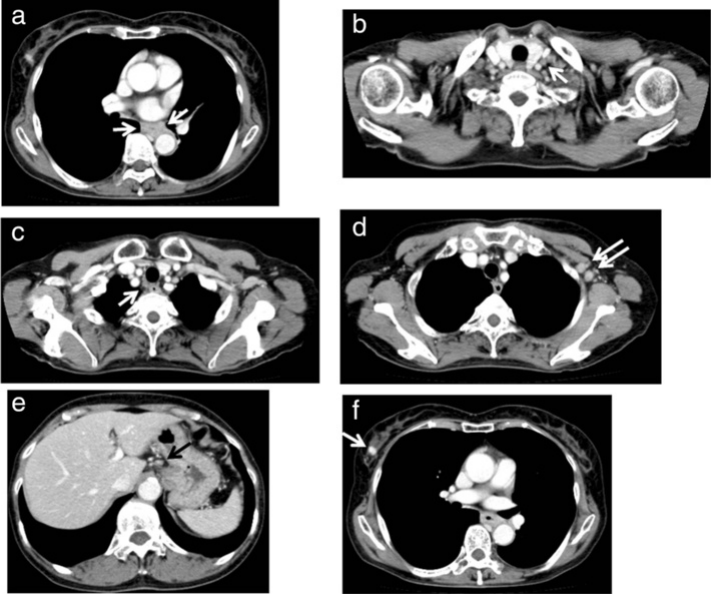
Chest and abdominal computed tomography at admission. **a** Wall thickening in the middle thoracic esophagus. **b** The left supraclavicular lymph node (7.0 mm in the short-axis plane). **c** The right recurrent nerve lymph node (7.0 mm in the short-axis plane). **d** Lymph node metastases in the left axillary space (13.8 and 14.7 mm in the short-axis plane). **e** Lymph node along the lesser curvature of the stomach (6.7 mm in the short-axis plane). **f** Mass of the right breast (maximum diameter of 16 mm)

A 64-year-old woman was admitted to our hospital with dysphagia in November 2012. Esophagogastroscopy revealed an ulcerated circumferential mass in the middle thoracic esophagus (Fig. 1a), and histopathological examination of the biopsy showed squamous cell carcinoma (Fig. 1b). Computed tomography (CT) revealed an esophageal tumor, right breast tumor, and two enlarged axillary lymph nodes on the left side. However, invasion of the esophageal tumor to adjacent organs was not observed (Fig. 2a). The breast tumor was 16 mm in diameter and was located at the lateral area of the right breast tissue (Fig. 2f). Two enlarged lymph nodes were observed in the left axillary space (13.8 and 14.7 mm in the short-axis plane) (Fig. 2d). All the other detectable lymph nodes (left supraclavicular node, right recurrent nerve node, and the node in the lesser curvature of the stomach) were less than 7.0 mm in size (Fig. 2b, c, e).

**Fig. 3:**
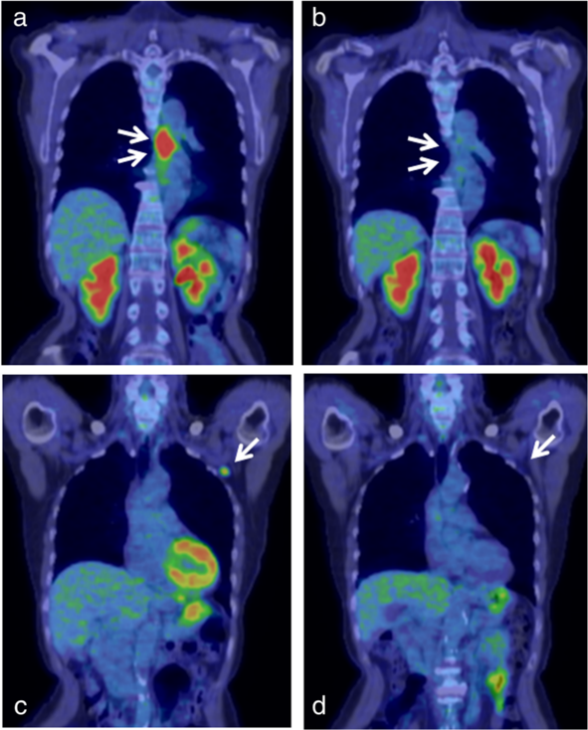
Positron emission tomography–computed tomography (PET–CT) findings. **a** PET–CT scan showing accumulation in the middle esophagus (standardized uptake value [SUV] max of 12.6). **b** PET–CT scan after four courses of chemotherapy with docetaxel, cisplatin, and 5-fluorouracil (DCF) showing the disappearance of the accumulation in the primary tumor. **c** PET–CT scan showing accumulation in the left axillary lymph node (SUV max of 3.6). **d** PET–CT scan after four courses of DCF treatment showing the disappearance of the accumulation in the left axillary lymph node

Fluorine-18 (18F) fluorodeoxyglucose (FDG) positron emission tomography–computed tomography (PET–CT) scan showed hypermetabolic lesions in the thoracic esophagus [standardized uptake value (SUV) max of 12.6], right breast (SUV max of 2.0), left axillary lymph nodes (SUV max of 3.6; Fig. 3a, c), and right supraclavicular region (SUV max of 3.4), which was not detected in the CT scan. FDG accumulation was not observed in the other nodes, including the lymph nodes detected in the chest and abdominal CT. Ultrasonographic examination revealed a solid mass measuring 1.0 cm × 0.9 cm in the upper outer quadrant of the right breast. Core needle biopsy from the tumor in the right breast revealed a scirrhous carcinoma, a subtype of invasive ductal carcinoma, that was positive for estrogen receptors (ERs) but negative for progesterone receptors (PgRs) and HER2/Neu. Fine-needle aspiration cytology from the left ALM did not provide definitive diagnosis of cancer cells. The clinical diagnosis was esophageal cancer (T3N0M1 stage IV according to the 7th edition of the Union for International Cancer Control classification) and synchronous right breast cancer (T1cN0M0 stage I). She was treated initially by triple chemotherapy with docetaxel, cisplatin, and 5-fluorouracil (DCF) as an induction chemotherapy for the esophageal cancer, with four courses over 4 weeks of docetaxel (60 mg/m^2^ on day 1), cisplatin (80 mg/m^2^ on day 1), and 5-fluorouracil (800 mg/m^2^ on days 1–5).

**Fig. 4:**
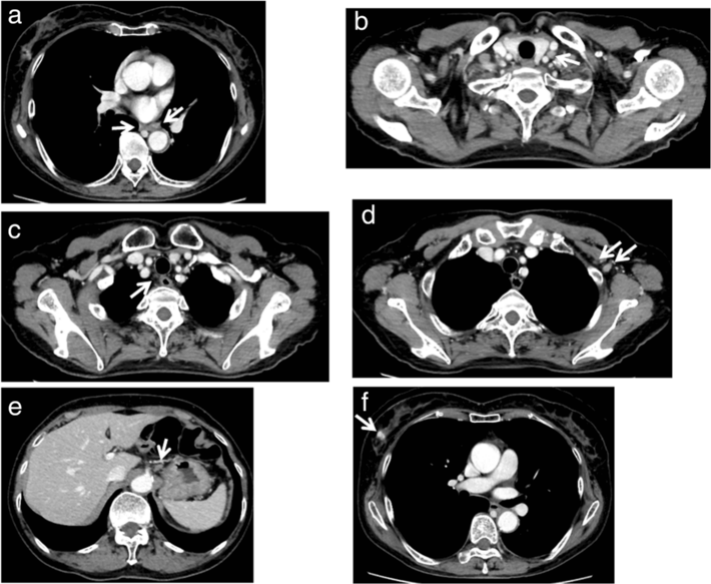
Chest and abdominal computed tomography after four courses of chemotherapy with docetaxel, cisplatin, and 5-fluorouracil. **a** The wall thickening in the middle thoracic esophagus had almost disappeared. **b** The size of the left supraclavicular lymph node did not change (8.0 mm in the short-axis plane). **c** The size of the right recurrent nerve lymph node had reduced (3.3 mm in the short-axis plane). **d** The size of the lymph node metastases in the left axillary space had reduced (9.3 and 2.1 mm in the short-axis plane). **e** The size of the lymph node along the lesser curvature of the stomach had reduced (5.7 mm in the short-axis plane). **f** The mass of the right breast had reduced in size (maximum diameter of 13.6 mm)

**Fig. 5:**
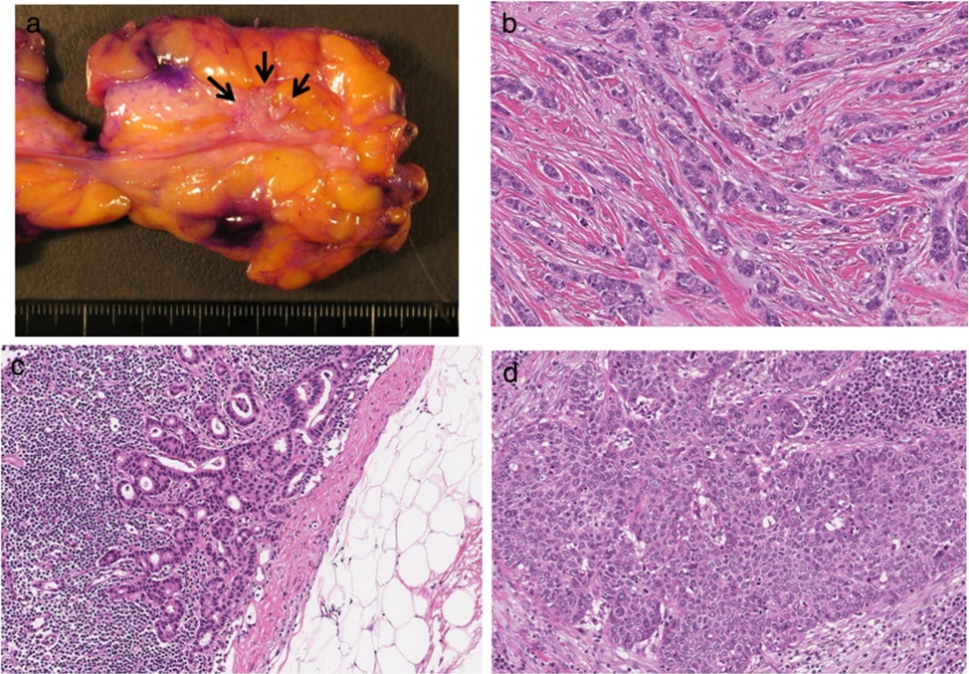
Macroscopic and microscopic findings of the resected specimen. **a** Surgical specimen of the right breast. **b** Pathological examination of a specimen from the breast showed scirrhous carcinoma, a grade II invasive ductal carcinoma (×100 magnification). **c** Pathological examination of a specimen from the right axillary lymph node showed metastatic cells from the invasive ductal carcinoma of the breast (×100 magnification). **d** Pathological examination of a specimen from the left axillary lymph node showed metastatic cells from the squamous cell carcinoma of the esophagus (×100 magnification)

Grade 3 leukopenia and neutropenia (according to the Common Terminology Criteria for Adverse Events Version 4.0) were observed during each course, but all other events were mild in severity. The therapeutic effect of the chemotherapy was evaluated after the four courses of DCF in April 2013. The primary lesion in the esophagus was markedly flattened, and only shallow ulcer scarring was observed by esophageal endoscopy (Fig. 1c). The biopsy from the ulcerative lesion revealed no malignancy. The chest CT scan showed that the left ALM and right breast cancer had both reduced in size (Fig. 4d, f). The PET–CT scan showed disappearance of the FDG accumulation in the primary tumor and left ALM (Fig. 3b, d) but no changes in the right breast cancer (SUV max 2.0). She underwent partial mastectomy of the right breast and bilateral axillary lymph node dissection. The histopathological diagnosis was as follows: invasive ductal carcinoma, scirrhous carcinoma, nuclear grade 2, positive for lymph node metastasis (1/16), ER: positive, PgR: negative, HER2/Neu: negative (1+ on immunohistochemistry), Ki67: <5, phenotype: luminal A, pTNM stage: T1cN1M0 stage IIA. One sentinel lymph node in the right axilla contained metastatic cells from the breast cancer (Fig. 5c). The chemotherapy was not histologically efficacious for either the right breast cancer or the right axillary lymph node (grade 0 according to The General Rules for Clinical and Pathological Recording of Breast Cancer, 17th edition) [8]. These findings suggest that DCF was not effective for the treatment of breast cancer in this case. In contrast, only one of the nine dissected lymph nodes from the left axilla contained metastatic cells from the squamous cell carcinoma of the esophagus (Fig. 5d). DCF treatment was shown to be slightly effective by histological evaluation of the left axillary metastatic lymph node (grade 1a according to the Japanese Classification of Esophageal Cancer, 10th edition) [9]. There were no complications associated with the chemotherapy during or after surgery. The patient was discharged from hospital 5 days after the surgery with no complications.

One month after the operation, she underwent chemoradiotherapy (CRT) for the esophageal cancer. The CRT regimen comprised cisplatin (70 mg/m^2^) on days 1 and 29, 5-fluorouracil (700 mg/m^2^) on days 1–4 and 29–32, and radiotherapy involving a total of 60 Gy in 30 fractions. The target fields of radiotherapy for esophageal cancer were the main tumor and regional lymph nodes, including the mediastinum and left supraclavicular nodes, and involved a total of 60 Gy. During chemotherapy, metastases were not detected in the right supraclavicular and abdominal regions; thus, these regions were excluded from the radiation field to preserve the bloodstream in the cervical esophagus and trachea, in consideration of salvage esophagectomy when recurrence of esophageal tumor would be observed in the future. Prophylactic irradiation after lymphadenectomy of the left axillary space was also simultaneously performed with a total dose of 50.4 Gy. Because of the prognosis of advanced esophageal cancer and the effectiveness of local control by an aromatase inhibitor, we decided to omit radiation therapy to conserve the breast.

Evaluation of the primary lesion in the esophagus after CRT using endoscopy revealed a complete response (Fig. 1d), and the patient is still relapse free 2 years after treatment. The patient was administered a common aromatase inhibitor, letrozole, as a hormonal therapy after mastectomy.

### Discussion

There have been few case reports of metastasis of the breast cancer to the esophagus and vice versa [10, 11] or metachronous esophageal cancer developing after radiation therapy for primary breast cancer [12, 13]. However, synchronous double cancer of the esophagus and breast is very rare, and only one case has been reported to our knowledge [4]. Natugoe et al. [1] reported that double cancer of the esophagus and breast was observed in only one patient in 157 cases of multiple primary cancers with esophageal squamous cell carcinoma, including synchronous and metachronous types. From the epidemiological perspective, the main reason for the low frequency of multiple cancers of the esophagus and breast is the gender difference for each cancer type. Esophageal squamous cell carcinoma occurs about six times more frequently in men than women [14], and the overwhelming majority of breast cancer occurs in women. Moreover, cancers in the upper aerodigestive tract, such as the head and neck, esophagus, stomach, and lung, may be commonly influenced by direct exposure of irritant substances by tobacco smoking and alcohol consumption. Although tobacco and alcohol are also risk factors for breast cancer, these irritants may have less influence on the mechanism of breast cancer development compared with that of the aerodigestive tract.

In our present case, histological diagnosis of the left ALM could not be obtained at the commencement of treatment. Both the ALM from the esophageal cancer and contralateral breast cancer were diagnosed as distant metastases, which are usually treated as systemic diseases. Incidences of contralateral ALM vary between 1.9 and 6 % for all breast cancers [6]. Morcos et al. [7] reported 21 patients with contralateral ALM; this type of metastasis is associated with tumors with aggressive pathological features, including locally advanced cancer (cT3/cT4: 95 %, stage III: 90 %), invasive ductal type (95 %), high grade (grade 3 carcinomas: 81 %), lymphovascular invasion (81 %), ER-receptor negativity (52 %), and HER2 overexpression (42 %). The breast cancer in our case was clinically diagnosed as T1cN0M0 (stage I); thus, we considered the left ALM to be a metastasis from the esophageal cancer.

ALM from esophageal cancer is rare [5]. Komatsu et al. reported four cases of ALM from esophageal cancer, and the incidence was 1.1 % in 361 esophageal cancer patients who underwent esophagectomy. In the report, all four patients had left ALM with ipsilateral left supraclavicular lymph node metastasis, and all ALMs were recognized as recurrences after locoregional treatment for esophageal cancer; two cases developed after esophagectomy and the other two after CRT for the primary lesions. This is the first report of ALM synchronously occurring with primary esophageal cancer. The mechanisms underlying metastasis to the axillary lymph nodes in esophageal cancer have not yet been elucidated. However, the theory of retrograde spread from the supraclavicular lymph nodes to the axillary nodes has been proposed in bronchogenic and lung carcinomas [15, 16]. Because all four cases of ALM reported by Komatsu et al. [5] had supraclavicular lymph node metastasis, the authors concluded that ALM from esophageal cancer could also be caused by retrograde flow owing to lymphatic blockade by the supraclavicular lymph node metastasis. In our present case, however, supraclavicular lymph node metastasis was not observed, although left ALMs were revealed by the CT and PET–CT scans. Bourgeosis et al. [17] reported the pattern and frequency of thoracic lymph duct drainage routes by visualization of the supradiaphragmatic lymph nodes using lymphoscintigraphy in 334 cases. In their results, supradiaphragmatic lymph nodes associated with the thoracic duct were demonstrated in 43.2 % of cases, with 10 % in the mediastinum, 41 % in the left supraclavicular/retroclavicular region, and 2.4 % in the right supraclavicular/retroclavicular region. Interestingly, in two cases (0.6 %), draining of the mediastinum and a chain of lymph nodes toward the left axilla was demonstrated without visualizing the supraclavicular nodes. Therefore, it is possible that our present case had a direct lymphoid route to the left axilla bypassing the left supraclavicular area.

Triplet chemotherapy with DCF has shown superiority to combined cisplatin and 5-fluorouracil in terms of overall survival for head and neck cancer as well as gastric cancer [18–20]. Recently, DCF has also been reported to be effective for esophageal cancer [21, 22], with several reports demonstrating a 53.7–62.5 % response rate of esophageal cancer to DCF; progressive disease was observed in 0–1.9 % [21, 22], and thus, DCF may be useful for neoadjuvant or induction therapy. Each drug in DCF has been widely used in chemotherapy for various cancers, and high efficacy of DCF has been shown in various cancers regardless of histological type, whether it is squamous cell carcinoma or adenocarcinoma. Therefore, DCF may be a suitable first-line therapy for multiple primary cancers of the esophagus and other organs with distant metastasis.

We selected induction DCF chemotherapy as the first-line therapy for this case of systemic disease of esophageal cancer. As the primary tumor size was markedly reduced after four courses of DCF and new lesions were not observed during approximately 5 months of chemotherapy, we assumed that this case of radical cure of cancer was due to the relatively localized disease. It is well known that the rate of radical cure from advanced esophageal cancer by systemic chemotherapy alone is quite low, and locoregional treatment is usually required to cure these patients. Considering that the patient was originally diagnosed with stage IV esophageal cancer with distant metastasis, we considered that CRT was preferable than surgery because of the concurrent use of chemotherapy with cisplatin and 5-fluorouracil as systemic therapy. We proposed both the treatments to the patient, and she selected CRT. Two courses of combination chemotherapy with cisplatin and 5-fluorouracil are usually used in standard CRT for esophageal cancer. To prevent irreversible adverse events, such as neuropathy and renal failure, due to the highly accumulative dose of cisplatin, CRT was followed by four cycles of DCF in this case. The total dose of cisplatin was 460 mg/m^2^, and no adverse events have been observed to date.

## Conclusions

Herein, we reported the successful treatment of synchronous double cancers of the esophagus with left ALM and right breast by combination therapy with chemotherapy, CRT, and surgery.

## Consent

Written informed consent was obtained from the patient for publication of this case report and the accompanying images. A copy of the written consent is available for review from the Editor-in-Chief of this journal.

## Abbreviations

ALM: axillary lymph node metastasis
CRT: chemoradiotherapy
CT: computed tomography
DCF: chemotherapy with docetaxel, cisplatin, and 5-fluorouracil
ERs: estrogen receptors
PET–CT: positron emission tomography–computed tomography
PgRs: progesterone receptors
SUV: standardized uptake value
